# The translation and psychometric assessment of the perception of empowerment in midwifery scale: the Persian version

**DOI:** 10.1186/s12913-020-05326-y

**Published:** 2020-05-26

**Authors:** Maryam Hajiesmaello, Nourossadat Kariman, Hamid Sharif Nia, Gity Ozgoli, Sepideh Hajian, Shahin Bazzazian, Tahereh Mokhtarian-Gilani

**Affiliations:** 1 Student Research Committee, Department of Midwifery and Reproductive Health, School of Nursing and Midwifery, Shahid Beheshti University of Medical Sciences, Tehran, Iran; 2 Midwifery and Reproductive Health Research Center, Department of Midwifery and Reproductive Health, School of Nursing and Midwifery, Shahid Beheshti University of Medical Sciences, Vali-e Asr Ave., Niayesh Intersection, Niayesh Complex, Tehran, Postal Code: 1985717443 Iran; 3 School of Nursing and Midwifery Amol, Mazandaran University of Medical Sciences, Sari, Iran

**Keywords:** Midwifery, Empowerment, Factor analysis, Psychometric assessment

## Abstract

**Background:**

A major part of midwifery care involves the empowerment of women and their families for the control of factors affecting their health. To this end, midwives should experience their own empowerment first. The present study was conducted to translate and determine the psychometric properties of the Perception of Empowerment in Midwifery Scale among Iranian midwives.

**Methods:**

A total of 380 people participated in this cross-sectional study. A demographic questionnaire and the 22-item Perception of Empowerment in Midwifery Scale were sent to the participants online. The validity of the scale and the analysis of its main components were carried out through exploratory factor analysis with Varimax rotation and confirmatory factor analysis. The reliability of the scale was assessed using the internal consistency method with Cronbach’s alpha coefficient, average inter-item correlation (AIC) and McDonald’s omega.

**Results:**

Seventeen scale items were retained after the exploratory factor analysis, and five factors were extracted, including “effective management”, “professional practice”, “authority”, “advocacy”, and “professional informant”, with factor loadings ranging from 0.489 to 0.899. The five latent factors explained 53.07% of the overall variance of the scale. The confirmatory factor analysis showed an acceptable goodness of fit. The internal consistency of the scale was confirmed with a Cronbach’s alpha above 0.7.

**Conclusion:**

The Persian version of the Perception of Empowerment in Midwifery Scale with 17 items has adequate reliability for midwives working in Iran. Given its appropriate psychometric properties, this scale is fit to be used among midwives in future studies.

## Background

Providing quality midwifery services is one of the main strategies for promoting maternal health. In some countries, the scope of midwives’ practices in healthcare has been confined by the healthcare system and some cultural barriers. Previous studies have shown that midwives have an effective role in providing cost-effective and high-quality maternal and neonatal care [1]. The factors affecting midwives’ decision to continue providing midwifery services or leave their job include the feeling of being supported by the managers, access to adequate resources, the development of relations with pregnant women and the experience of feeling empowered in and in control of their job [2]. The empowerment of midwives is a common goal among this group. Moreover, an important part of midwifery care is the empowerment of women and their families for controlling the factors affecting their health [3]. Meanwhile, if the empowerment of women is a responsibility of midwives, this group should first experience the empowerment of themselves [4].

The definition of the concept of empowerment and its perception vary depending on the cultural and social background and the theoretical approach used [5]. Empowerment means designing an organizational structure in which people are ready to accept further responsibilities while managing themselves. In other words, empowerment is a process in which the manager helps the personnel find the ability required for independent decision-making. This process affects not only individuals’ performances, but also their personality [6]. In nursing articles, empowerment is used as an umbrella concept to describe the elements of professional development and growth [7]. In an organizational context, empowerment has two theoretical orientations: Structural empowerment and psychological empowerment. Structural empowerment means the ability to equip resources, achieve goals by access to information, support and resources and opportunities for learning and development. Psychological empowerment refers to the individual’s psychological response to empowerment in relation to his/her work conditions [7].

Many studies confirm the importance of the empowerment of healthcare personnel. For example, the empowerment of nurses and its subsequent feeling of being in control in the workplace are necessary for a variety of reasons, and the lack of empowerment in nursing worsens job dissatisfaction in this group and paves the way for occupational burnout [8]. It also renders the efforts to correct clinical performance and innovation in patient care futile [9]. Nonetheless, the concept of empowerment may be different in midwifery due to the unique aspects of the midwifery profession, including authority. The International Confederation of Midwives (ICM) proposes midwifery as a profession of responsibility and accountability that begets the participation of women in providing their own gender with support and care [10]. Studies suggest that the most important conditions for the empowerment of midwives include control, support, diagnosis and identification and midwifery skills [11]. Today, many midwives increasingly feel that their professional skills have been taken away from them by other members of the medical field [12]. Yet, issues such as support on the part of the managers and having a clear definition of the roles and job descriptions can make midwives more empowered [11]. The Perception of Empowerment in Midwifery Scale (PEMS) is a tool for quantifying the criteria and conditions that are important for midwives’ perception of empowerment. This questionnaire has been developed to assess midwives’ perception of their level of empowerment with 22 items in three factors, namely “autonomous practice”, “effective management” and “women-centered practice”, and has been translated into various languages, and its reliability has been confirmed in several countries, including Norway, Portugal, Turkey, and New Zealand [13–17].

Midwifery has been taught at the bachelor’s degree level in Iran (i.e. directly upon the students’ entrance into university or college) in accordance with the international standards and has a history of about 100 years in this country [18]. Nevertheless, certain problems have limited the scope of work in this profession, including the lack of authority in providing maternal services, the refusal of insurance institutions and organizations to draw contracts with midwives and the absence of a suitable job market [19]. There are currently around 50,000 midwifery graduates with bachelor’s degrees and some with master’s degrees and PhDs in Iran, but their capacities are not entirely taken advantage of, and about 35,000 of them are not working in their own academic field, even though the ratio of midwives per 1000 births is lower in Iran than the global standards, and is about 12 per 1000 births [19, 20]. For the past decades, Iranian midwives have been independently providing care to pregnant women during their stages of pregnancy, labor, childbirth and postpartum. Over the last three decades, however, care provision to low-risk pregnant women has moved to the territory of medicine due to the physician dominance in the Iranian healthcare system, and midwives have had but a fading role in performing natural childbirth for mothers with low-risk pregnancy [20]. In general, 48% of all births are by cesarean section; in the private sector, this rate reaches 87% [21].

Following the Health Development Plan in Iran that began in 2014, policies were adopted to promote natural childbirth, including free-of-charge delivery in public hospitals, holding prenatal classes for mothers by midwives and holding physiologic birth workshops, and empower midwives; however, midwives continue to face limitations in providing midwifery services, especially in natural birth procedures. In teaching hospitals, natural childbirth is performed by female residents, and midwives have a low-profile role in providing maternal care during labor and childbirth. In non-teaching hospitals, midwives are not allowed to independently manage natural childbirths for low-risk women and can only perform natural childbirths under the supervision of specialists. In truth, gynecologists and obstetricians are responsible for the majority of childbirths in hospitals [20]. The evaluation of midwives’ perception of empowerment in Iran is therefore particularly important. The present study was designed and conducted to translate PEMS into Persian for the first time and assess its psychometric properties in Iranian midwives so as to offer a standard and reliable tool consistent with the dominant culture and health system of Iran that enables further research on important work-related factors involved in the empowerment of midwives and facilitates the empowerment of Iranian midwives.

## Methods

The present descriptive cross-sectional study was conducted to determine the psychometric properties of the Persian version of PEMS in Iranian midwives. The study population consisted of midwives providing care to women during pregnancy and childbirth. The study inclusion criteria consisted of having either a bachelor’s degree, master’s degree or PhD in midwifery, being employed at the time of the study and having a minimum of 1 year’s work experience. Working in fields other than pregnancy and childbirth care was among the study exclusion criteria. The tool used was a two-part online questionnaire that was sent to 2000 midwives who were members of professional groups in social networks. The first part dealt with demographic details such as age, marital status, qualification, number of years in service and workplace. The second part consisted of the Perception of Empowerment in Midwifery Scale, developed by Anne Matthews in Ireland, with confirmed validity and reliability [13]. This questionnaire contains 22 items in three factors, including “autonomous practice”, “effective management” and “women-centered practice”, each with six items, plus four additional items (i.e. items 11, 14, 15 and 19) that have not been included in any of the factors: “I am adequately educated to perform my role”, “I do not know what my scope of practice is”, “I am accountable for my practice” and “I do not have access to adequate resources for staff education and training”. Since the importance of these items for the empowerment of midwives has previously been strongly confirmed, they were not excluded by the author, who recommended further exploratory analyses with new data. To prevent errors in responding, a number of items were designed negatively, with reverse scoring compared to the other items. The responses consisted of a 5-point Likert scale including ‘strongly disagree’, ‘disagree’, ‘neither agree nor disagree’, ‘agree’ and ‘strongly agree’. In the present study, “strongly agree” scored the highest (5 points) and “strongly disagree” the lowest (1 point) for each item. The mean score in each factor was also calculated from 1 to 5 points, with the median being 3. The total score of the scale was calculated as the sum of the scores obtained in each factor, and higher scores indicated the highest perception of empowerment and lower scores indicated the lowest perception. This scoring system was adopted to simplify the interpretation of the scores, but is the exact reverse of the scoring system adopted in the original scale. Nevertheless, the adopted system concurs with the method of scoring and score interpretation used in other studies on the subject [14, 16, 17].

The present study was conducted in two stages. The first stage was translation, which began after obtaining written permission from the original designer of PEMS. For this purpose, the questionnaire was translated into Persian by two experienced translators, and then the two translations were combined and the best translation for each item was selected. The Persian questionnaire was then translated back into English by two translators unfamiliar with the original questionnaire, and after the review and integration of these two translations, the new translated English version was sent to the author of the original PEMS and modifications were made to it according to the author’s views. The second stage was concerned with determining the validity of the scale. To this end, the face validity (quantitative and qualitative), content validity (quantitative and qualitative) and construct validity of the items were measured. In the qualitative face validity, the views expressed by the target group (i.e. midwives) about the level of difficulty, relevancy and ambiguity of the items were used and necessary modifications were made to the items to make them easily comprehensible. In the quantitative method, the items’ impact score was calculated to eliminate the inappropriate items and determine the importance of each item. Items with an impact score > 1.5 were retained for further analysis. To assess the qualitative content validity, the views expressed by ten midwifery professors were used to make the necessary modifications, and the Content Validity Ratio (CVR) and Content Validity Index (CVI) were measured for the quantitative content validity assessment.

The construct validity of the scale was assessed using exploratory and confirmatory factor analyses. The construct validity of PEMS was assessed by the Maximum-Likelihood Estimation (MLE) method with varimax rotation. Sample adequacy was estimated through the Kaiser-Meyer-Olkin (KMO) index and Bartlett’s test. KMO values of 0.7–0.8 and 0.8–0.9 were interpreted as good and excellent, respectively. The sample size for factor analysis was estimated using the rule of thumb, and 380 was considered adequate for this study.

Items were allocated to a latent factor if they had a factor loading of almost 0.3, as estimated by the following formula: *CV = 5.152*÷ *√ (n – 2)*, where *CV* is the number of extractable factors and *n* the sample size. The number of latent factors was estimated using Horn’s parallel analysis [22]. For assessing the structural factors, CFA was performed using the MLE method and the most common goodness of fit indices. The model fit was assessed based on the Root Mean Square Error of Approximation (RMSEA),, Comparative Fit Index (CFI), Normed Fit Index (NFI), Goodness of Fit Index (GFI), and Adjusted Goodness of Fit Index (AGFI).

The reliability of the scale was assessed by the internal consistency method using Cronbach’s alpha coefficient, Average Inter-item Correlation (AIC) and McDonald’s omega. All the data analyses were performed in SPSS Amos 24 and the SPSS R-Menu 2.0.

## Results

A total of 411 responses with a participation rate of 20% were received. A total of 31 candidates were excluded for having less than 1 year’s work experience or not being employed as a midwife at the time of the study, and 380 eligible midwives participated in the study, with a mean age of 32.7 (8.5) years, age range of 23–67 years and mean number of years in service 8.5 (7.9), ranging from 1 to 47 years. Table 1 presents the demographic details of the participants. Once the qualitative face validity was assessed, one item (item 3) was modified based on the midwives’ views, because some midwives did not have a proper understanding of the concept of midwife-led practice. In the quantitative validity assessment stage, all the items had impact scores > 1.5 (ranging from 2.5 to 4.8) and were therefore retained. The views expressed by the professors and experts were implemented after the qualitative content validity assessment stage. Lawshe’s method was used to determine the quantitative content validity of the scale. This method was first proposed in 1975 and its application soon became widespread. In this method, the Content Validity Ratio (CVR) is determined based on the experts’ views, who are asked to divide each of the items into three categories, including “Essential”, “Useful but not essential”, and “Not essential”, and CVR is then calculated using the following formula: CVR= $$ \frac{Ne-\raisebox{1ex}{$N$}\!\left/ \!\raisebox{-1ex}{$2$}\right.}{\raisebox{1ex}{$N$}\!\left/ \!\raisebox{-1ex}{$2$}\right.} $$ (where *Ne* is the number of experts who have considered an item essential and *N* is the total number of experts) [23]. CVR is a number between − 1 and + 1., and CVR > 0 indicates that at least 50% of the experts have considered a given item essential. The minimum acceptable CVR is determined according to Lawshe’s table. Items with CVR less than that stated in the table for the given number of experts are unacceptable and should be eliminated from the scale [24]. In the present study, CVR was found as 0.91 (0.13) for the entire scale and ranged from 0.6 to 1 for each item, and since, according to Lawshe’s table and based on the number of experts (*n* = 10), items with a value > 0.6 were acceptable (Lawshe, 1975), all the questionnaire items were retained.

**Table 1:** Participants' demographic details

	Number	Percentage
Age (years)		
23-30	208	54.7
31-40	97	25.5
41-50	62	16.3
Older than 50	13	3.4
Marital status		
Single	138	36.3
Married	242	63.7
Qualifications		
Bachelor's degree	308	81
Master's degree	60	15.8
PhD	12	3.2
Years of service		
1-2	78	20.5
3-10	187	49.2
11-20	70	18.4
21-30	35	9.2
More than 30	2	0.5
Missing	8	1/2
Place of work		
Public hospital Private hospital	198 28	52.1 7.4
Private surgery	13	3.4
Health centers	138	36.3
Missing	3	0.8

The CVI was calculated as 0.93 (0.04) for the entire scale, ranging from 0.83 to 1 for each item, and since CVI > 0.79 is considered appropriate [25], all the items in the scale were finally accepted.

This study also examined the adequacy of sampling for factor analysis and reported the KMO index as 0.797. Bartlett’s test of sphericity was also performed using the Chi-square statistic, which was reported as 2006.376, thus proving significant at *P* < 0.001. The minimum conditions required for conducting exploratory factor analyses were therefore met.

In this research, based on the Exploratory Factor Analysis (EFA) results proposed by Matthews et al. in 2009, a Confirmatory Factor Analysis (CFA) was carried out first, the results of which are presented in Table 3. The CFA results and fit indices were inappropriate; therefore, another EFA was performed in this stage on the collected data. Subsequently, a CFA was performed based on the results of this EFA. Table 3 presents the CFA results for the purpose of comparison with the CFA results pertaining to the original scale. As observed, the indices without modification confirm the fit of the five-factor model.

**Fig. 1:**
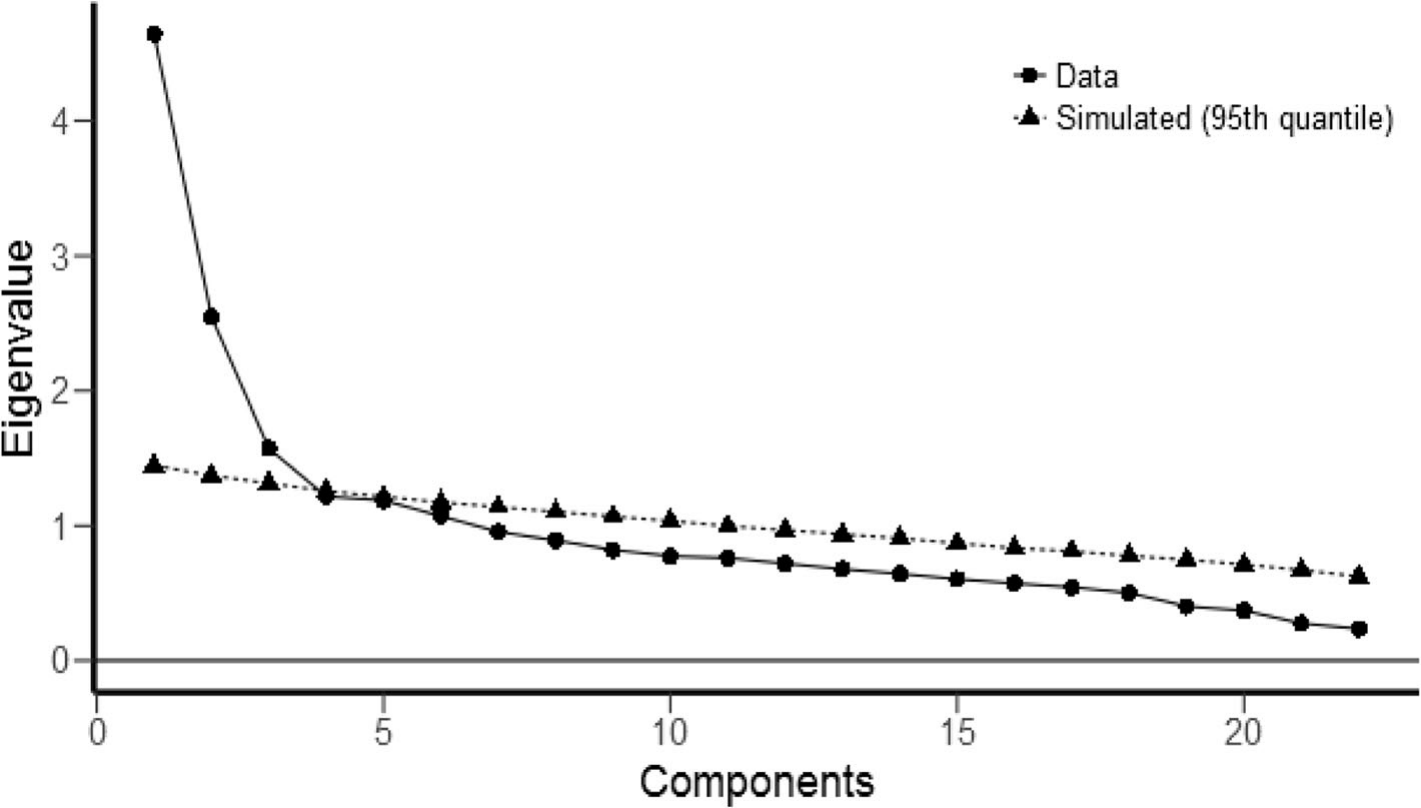
The scree plot of the extracted factors

**Fig. 2:**
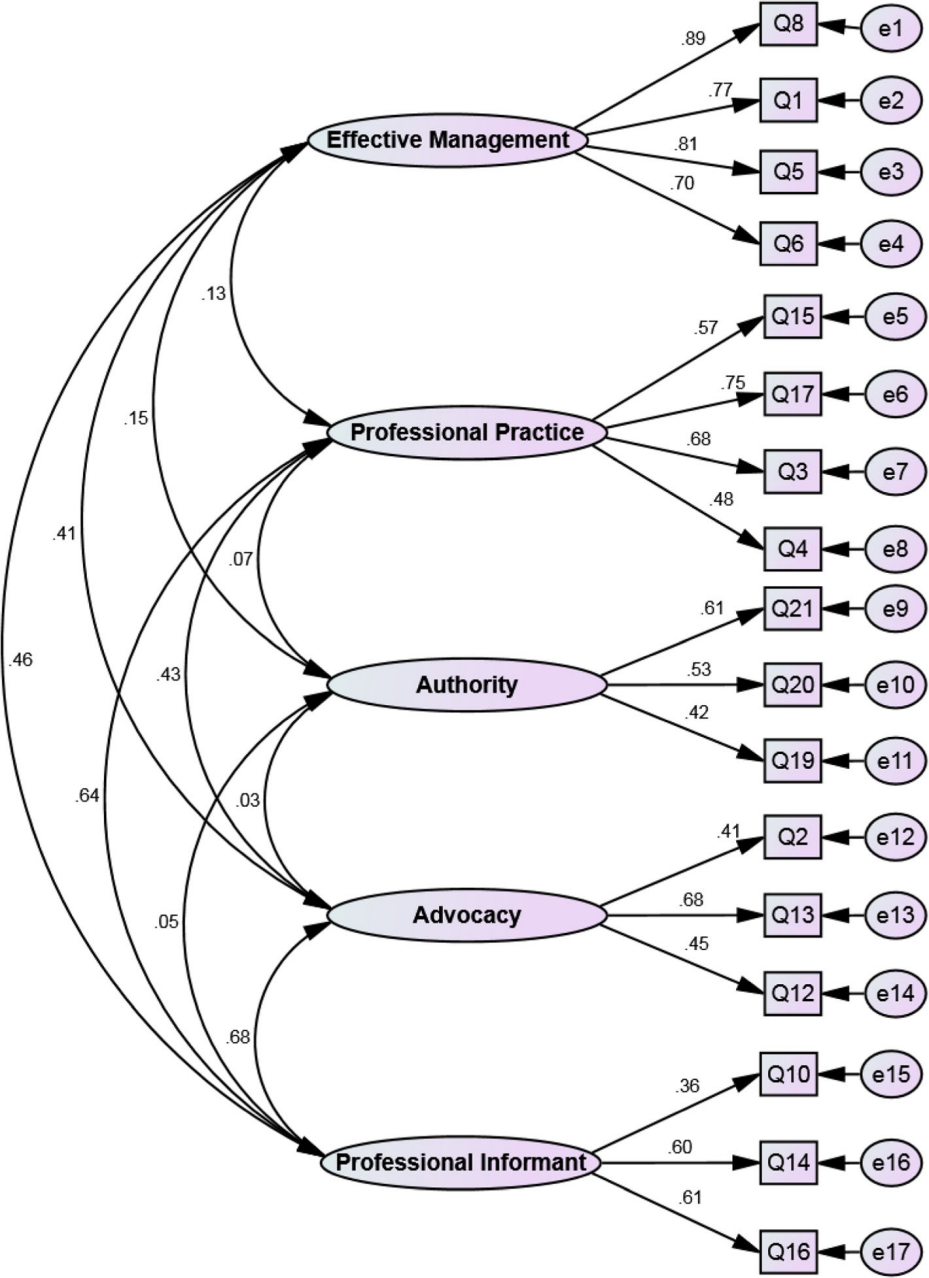
The final structure of the model of the Perception of Empowerment in Midwifery Scale among Iranians

The latent factors were extracted by EFA and varimax rotation. Given that Eigenvalues > 1 and based on the Scree plot (Fig. 1), five factors were extracted, which explained 53.07% of the total variance in the variables. After this analysis, 17 of the scale items were retained. Factor one was named ‘effective management’, with items 1, 5, 6 and 8. Factor two was named ‘professional practice’, with items 3, 4, 15 and 17. Factor three was named ‘authority’, with items 19, 20 and 21. Factor four was named ‘advocacy’, with items 2, 12 and 13. Factor five was named ‘professional informant’, with items 10, 14 and 16 (Table 2) (Fig. 2). The goodness of fit of the final factor structure of the 17-item questionnaire was assessed using the Chi-square goodness of fit test with confirmatory factor analysis. Next, the model fit of the other indices was assessed, and the results obtained confirmed the suitable fit of the final model (Table 3). The Cronbach’s alpha and McDonald’s omega of the five extracted factors were acceptable (0.7<). Also, as shown in Table 2, the AIC was between 0.346 and 0.626 for all the factors, which is considered good [26].

**Table 2:** The exploratory factors extracted from the PEMS

Factor	Q_n_. Item	Factor loading	h^2^	%Variance	Eigenvalue	Internal consistency	Mean SD
Effective management	8. I do not have a supportive manager	0.899	0.809	**16.54**	**2.818**	**α (CI95%): 0.870 (0.848 to 0.890)** **Ω: 0.873** **AIC: .0.626**	**3.16** **0.89**
	1. I have the back-up of my manager	0.862	0.712				
	5. I am valued by my manager	0.830	0.752				
	6. I am not recognized for my contribution to the care of birthing women by my manager	0.757	0.616				
Professional Practice	15. I am accountable for my practice	0.779	0.560	**9.97**	**1.695**	**α (CI95%): 0.709 (0.658 to 0.754)** **Ω: 0.719** **AIC: 0.384**	**4.36** **0.43**
	17. I have control over my practice	0.756	0.630				
	3. I am involved in midwife-led practice	0.719	0.572				
	4. I do not have the skills required to carry out my role	0.699	0.557				
Authority	21. I am not listened to by members of the multidisciplinary team	0.779	0.611	**8.97**	**1.525**	**α (CI95%): 0.719 (0.715 to 0.721)** **Ω: 0.728** **AIC: 0.365**	**3.1** **0.73**
	20. I have autonomy in my practice	0.745	0.550				
	19. I do not have access to adequate resources for staff education and training	0.603	0.425				
Advocacy	2. I am an advocate for birthing women	0.863	0.659	**10.11**	**1.720**	**α (CI95%): 0.736 (0.702 to 0.738)** **Ω: 0.740** **AIC: 0.346**	**3.61** **0.66**
	13. I am able to say no when I judge it to be necessary	0.697	0.590				
	12. I have support from my colleagues	0.489	0.365				
Professional informant	10. I am not informed about changes in my organization that will affect my practice	0.846	0.670	**7.48**	**1.272**	**α (CI95%): 0.736 (0.732 to 0.741)** **Ω: 0.745** **AIC: 0.380**	**3.46** **0.71**
	14. I do not know what my scope of practice is	0.552	0.514				
	16. I am recognized as a professional by the medical profession	0.502	0.520

**Table 3:** The fit model indices of CFA of PEMS

Indices^a^ Model	χ^2^	df	*P* value	CMIN/DF	RMSEA	PCFI	PNFI	IFI	CFI	AGFI	GFI
**Original scale** **(3 factors)**	398.661	132	< .001	3.020	0.073	0.714	0.660	0.830	0.827		
**Modified model** **(5 factors)**	172.383	109	< .001	1.581	.039	0.768	0.718	0.959	0.958	0.931	0.951

^a^Acceptable values are as follows: > 0.5 for PNFI, PCFI, AGFI; > 0.9 for CFI and IFI; > 0.08 for RMSEA; and > 0.5 for CMIN/DF

## Discussion

Validity and reliability are usually the key quality indicators of measurement instruments. In simple words, validity indicates the rigor and accuracy of a tool and reliability shows its stability [27]. The present study was conducted to assess the validity, reliability and factor structure of the Persian version of PEMS and present a reliable tool in Persian language that is compatible with the culture and work conditions in Iran’s healthcare system. PEMS is specifically designed for midwives and has been developed according to the views of midwives about what empowerment is for them and is thus a useful tool for assessing midwives’ perception of their own performance and work environment. All the items of the questionnaire were translated in a simple, clear and relevant manner and the Persian version obtained acceptable face and content validities.

According to the results of the exploratory factor analysis, the 17 items of the questionnaire were placed under five factors based on the factors extracted, including Eigenvalue, cumulative variance, the variance of each factor and the scree plot assessment. Since, in the naming process, the most emphasis has to be on the three or four variables with the highest loading, five factors were accordingly named. Meanwhile, one cannot expect that all the variables loaded onto one factor be conceptually consistent or that the name chosen for one factor reflect the meaning of all the items exactly [28]. These five factors were named as follows: “Effective management” (four item), “professional practice” (four items), “authority” (three items), “advocacy” (three items) and “professional informant” (three items).

The confirmatory factor analysis was conducted to assess the structure of the final model of the tool with the five extracted factors. In general, indicators used for assessing a model fit are divided into three general groups, including absolute fit, comparative fit and parsimonious fit, and it would be best to report at least one indicator from each group [29]. In the present study, RMSEA =0.039, CMIN/DF = 1/581 and IFI =0.959, which indicate an acceptable fit. The original version had confirmed three factors with six items in each [13], and this inconsistency in the number of factors and type of items in each factor could be attributed to the cultural differences, different work conditions and respondents’ varying perceptions of the questionnaire items. In a study conducted in New Zealand to determine the validity of PEMS, the exploratory factor analysis produced four factors, including autonomy and empowerment (4 items), manager support, professional support, skills and resources (5 items each), and items 10, 13 and 15 were excluded [17].

The scoring in the original version of the scale was in such a way that the mean score in each factor varied from 1 to 5 points. In the present study, the reverse order was used for scoring the items in order to simplify the perception and interpretation of the scores; that is, the mean scores indicate very low, low, moderate, high and very high perceptions of empowerment as we move from 1 to 5 points. In the original version of PEMS, the total score of the scale is calculated as the sum of the scores of the three factors, and the lowest possible score is 3 and the highest 15, and 9 is the median score. In the present study, following the scoring method used in the original version of PEMS, the mean total score was calculated as the sum of the scores in the five factors. The lowest possible score was 5 and the highest 25, and 15 was defined as the median. The total score was divided as follows: Scores 21–25 indicated a very high perception of empowerment in the midwives, 16–20 indicated a high perception, 15 moderate, 11–14 low and 5–10 very low perception of empowerment.

Accordingly, based on the present findings, the perception of empowerment is high in 76.8% of midwives and poor and very poor in 17.4%. The mean score obtained in the four factors was in the moderate range in the present study (mean effective management score = 3.16 (0.87), mean authority score = 3.1 (0.73), mean advocacy score = 3.61 (0.66) and mean professional informant score = 3.46 (0.71)), but the mean scores obtained for “professional practice” were 4.36(0.43), which show high perceived empowerment. The mean overall score obtained was 17.71 (2.05), confirming the high perception of empowerment among Iranian midwives in the study.

Since the number of factors obtained and the items related to each factor are different in the present study than the other studies, and since five items were eliminated, the results cannot be compared with those of the other studies. Nonetheless, according to the mean score of the items in the three factors obtained in Ozturk’s study [16], there are similarities between Turkey and Iran, since the midwives in both countries had a moderate perception of empowerment in the “support and management” (Turkey: 3.42 ± 0.57 and Iran: 3.51 ± 0.54) and “resource” (Turkey 3.30 ± 0.51 and Iran: 3.70 ± 0.47) factors and a high perception of the “skill” factor (Turkey: 4.05 ± 0.91 and Iran: 4.10 ± 0.44). Meanwhile, based on the results of the study conducted by Lukasse & Pajalic in Norwegian midwives in three factors [14], the mean scores obtained for the “autonomous professional role” (4.00 ± 0.47) and “equipped for practice” (4.50 ± 0.46) factors were high, while the corresponding mean scores obtained by Iranian midwives were moderate (3.82 ± 0.41 and 3.83 ± 0.54, respectively). This difference could be due to the differences in the health and education systems and health policy-making between the countries.

## Conclusion

Based on the results obtained, the Persian version of PEMS was found to have an acceptable validity and reliability in the study population with the finalized factors and items. The small number of items in this scale makes it easier to use and it also does not require much time to be completed. Moreover, given the importance of the role of midwifery education institutions and managers in the empowerment of midwives, this scale can be more extensively used to understand the adequacy of these two factors.

## Data Availability

The datasets used during the present study can be accessed from the corresponding author on reasonable request.

## Abbreviations

PEMS: Empowerment in midwifery scale
AIC: Average interitem correlation
ICM: International confederation of midwives
CVR: Content validity ratio
CVI: Content validity index;
KMO: Kaiser-meyer-olkin
MLE: Maximum-likelihood exploratory
GFI: Goodness of fit index
RMSEA: Root mean square error of approximation
CMIN/DF: Chi-square mean/degree of freedom
IFI: Incremental fit index
