# Correction to: The translation and psychometric assessment of the perception of empowerment in midwifery scale: the Persian version

**DOI:** 10.1186/s12913-020-05519-5

**Published:** 2020-07-13

**Authors:** Maryam Hajiesmaello, Nourossadat Kariman, Hamid Sharif Nia, Giti Ozgoli, Sepideh Hajian, Shahin Bazzazian, Tahereh Mokhtarian-Gilani

**Affiliations:** 1 Student Research Committee, Department of Midwifery and Reproductive Health, School of Nursing and Midwifery, Shahid Beheshti University of Medical Sciences, Tehran, Iran; 2 Midwifery and Reproductive Health Research Center, Department of Midwifery and Reproductive Health, School of Nursing and Midwifery, Shahid Beheshti University of Medical Sciences, Vali-e Asr Ave., Niayesh Intersection, Niayesh Complex, Tehran, Postal Code: 1985717443 Iran; 3 School of Nursing and Midwifery Amol, Mazandaran University of Medical Sciences, Sari, Iran

**Correction to: BMC Health Services Research (2020) 20:466**

**https://doi.org/10.1186/s12913-020-05326-y**

Following publication of the original article [1], the authors identified an error in the author name of Giti Ozgoli.

The incorrect author name is: Gity Ozgoli

The correct author name is: Giti Ozgoli
